# IMRT and carbon ion boost for malignant salivary gland tumors: interim analysis of the COSMIC trial

**DOI:** 10.1186/1471-2407-12-163

**Published:** 2012-05-02

**Authors:** Alexandra D Jensen, Anna V Nikoghosyan, Karen Lossner, Klaus K Herfarth, Jürgen Debus, Marc W Münter

**Affiliations:** 1 Dept of Radiation Oncology, University of Heidelberg, INF 400, 69120, Heidelberg, Germany

## Abstract

**Background:**

The COSMIC trial is designed to evaluate toxicity in dose-escalated treatment with intensity-modulated radiotherapy (IMRT) and carbon ion boost for malignant salivary gland tumors (MSGT) of the head and neck including patients with inoperable/ incompletely resected MSGTs (R2-group) and completely resected tumors plus involved margins or perineural spread (R1-group).

**Methods:**

COSMIC is a prospective phase II trial of IMRT (25 × 2 Gy) and carbon ion boost (8 × 3 GyE). Primary endpoint is mucositis CTC°III, secondary endpoints are local control, progression-free survival, and toxicity. Evaluation of disease response is carried out according to the Response Evaluation Criteria in Solid Tumors (RECIST); toxicity is assessed using NCI CTC v 3.0.

**Results:**

Twenty-nine patients were recruited from 07/2010 to 04/2011, all patients have at least completed first follow-up. Sixteen patients were treated in the R2-group, 13 in the R1-group. All treatments were completed as planned and well tolerated, mucositis CTC grade III was 25% (R2) and 15.4% (R1), no dysphagia CTC grade III was observed, no feeding tubes were necessary. Side-effects rapidly resolved, only 4 patients (13.8%) reported xerostomia grade II at first follow-up. Overall response rate (complete and partial response) according to RECIST in the R2-group is 68.8% at 6–8 weeks post treatment, all patients within this group showed radiological signs of treatment response.

**Conclusion:**

No unexpected toxicity was observed, mucositis rates and other side effects do not differ between patients with visible residual tumor and macroscopically completely resected tumors. Initial treatment response is promising though longer follow-up is needed to assess local control.

**Trial registration:**

Clinical trial identifier NCT 01154270

## Background

Local control for malignant salivary gland tumors (MSGT) remains a challenge.

While high-precision techniques such as intensity-modulated radiotherapy (IMRT) and fractionated stereotactic radiotherapy (FSRT) could already improve local control as compared to conventional RT techniques and achieve 3-year PFS rates around 38% [1], significant improvements were only seen by application of particle therapy. So far, the highest local control rates at 75 – 100% [2,3] were achieved by neutron radiation albeit at the cost of significant late toxicity. Heavy ion therapy using carbon ions however, so far only showed a mild toxicity profile even in dose escalation and hypofractionation [4].

The German carbon ion pilot project succeeded to establish a mixed beam regimen consisting of intensity-modulated radiotherapy (IMRT) and carbon ion boost for adenoid cystic carcinoma: with 78% at 4 years, local control rates were in the range of results formerly achieved by neutron therapy but without the dreaded late toxicity [5]. A recent update of all patients with adenoid cystic carcinoma treated with this regimen between 1997 and 2008 supports these findings with consistently low treatment-related side effects [6]. These results in turn led to the acceptance of this regimen as the standard treatment and method of choice for adenoid cystic carcinoma in Germany.

Doses recommended for treatment of malignant salivary gland tumors are high [7-9], due to the possibility to apply higher doses at so far mild side effects, charged-particle therapy does offer the promise of improved results for all types of malignant salivary gland cancers whether in the definitive or adjuvant setting.

Whereas the initial carbon ion project only included patients with inoperable or incompletely resected adenoid cystic carcinoma, the COSMIC trial was designed to investigate efficacy and toxicity of combination therapy as IMRT plus carbon ion boost also for all types of MSGTs as well as macroscopically completely resected tumors with risk factors such as R1-resections or perineural spread. As patient accrual is much faster than expected in this rare disease an interim analysis seems warranted.

## Methods

### Patients

Patients with pathologically confirmed malignant salivary gland tumor of the head and neck are eligible for this trial. Patients may be inoperable and receive treatment as definitive radiotherapy or have received surgery for their primary tumor with either macroscopic (R2) or microscopic (R1) residue and/ or perineural invasion. Age between 18 and 80 years, Karnofsky performance score of >70% were also required. Exclusion criteria were prior chemotherapy or radiotherapy [10].

Work-up included complete panendoscopy, diagnostic CT scans of the neck and chest and abdominal ultrasound. In the absence of contraindications, all patients receive diagnostic MRI scans for treatment planning and follow-up.

The trial was reviewed and approved by the University of Heidelberg Medical School Ethics Committee; informed consent is obtained from all patients prior to inclusion.

### Radiotherapy

#### Immobilisation/ planning examinations

Patients are immobilized using individual thermoplastic head masks incl. shoulder fixation (HeadStep®, ITV). Planning examinations consist of a planning CT scan (3 mm slice thickness) with the patient positioned in the individual fixation device and contrast-enhanced MRI for 3D image correlation.

#### Target volumes/ dose prescription and constraints

CTV1 (carbon ion boost) includes the macroscopic tumour/ prior tumour bed with special focus on the R1-area as well as respective neural pathways to the base of skull. For tumors of the parotid gland, the whole former parotid area is also included in the CTV1. In order to avoid potential late effects, the mandibular joint is kept outside the CTV1 whenever reasonably possible. PTV1 consists of a 3 mm margin around the CTV1 but does not extend into critical organs at risk (i.e. brain stem, spinal cord). Twenty-four GyE carbon ions in 3 GyE per fraction (5 fractions per week) are prescribed to the CTV1 as an upfront boost, the CTV1 should be covered with the 95% prescription isodose.

CTV2 includes CTV1 with safety margins along typical pathways of spread. Only ipsilateral nodal levels (II and III) are included, however, in case the primary tumour is located at or crossing midline, these are bilaterally included. In case of nodal metastases, additional nodal levels are covered as indicated. CTV2 needs to encompass the complete surgical operational area and accounts for set-up variations, hence corresponds to the PTV2 (CTV2 = PTV2). Should the primary tumour be located within the parotid gland, also the parotid duct needs to be within the CTV2. The CTV2 is prescribed 50 Gy IMRT in 25 fractions (5 fractions per week), coverage with at least the 90% prescription isodose needs to be aimed for.

Summation plans are evaluated according to the following criteria: < 20% of the CTV1 should receive ≥ 110% of the prescribed dose, <5% of CTV1 or CTV2 should receive ≤ 90% of the prescribed dose, and <2% or 2 cc of tissue outside the CTVs should receive ≥ 110% of the prescribed dose to the CTV1. In addition, the following normal tissue constraints were used in evaluation of the summation plan (carbon ion **and** photon IMRT) plan at standard fractionation (2 Gy/ fraction).

· Spinal cord: the dose to any point within the spinal cord should not exceed 50 Gy to any volume larger than 0.03 cc.

· Brain stem: the tolerated dose is 54 Gy; maximum tolerated dose in volumes of ≤ 1 cc : 60 Gy.

· Optic chiasm/ optic nerves: maximum dose to these structures should be ≤54 Gy, in case this dose limit cannot be kept without compromising target volume coverage, these issues were discussed with the patient and decisions made accordingly.

· Eyes: maximum doses ≤45 Gy to the posterior bulb/ retina; doses to the whole eye were reduced as low as reasonably achievable without compromising target volume coverage

· Parotid glands: mean dose to at least one gland below 26 Gy; alternatively at least 20 cc of the combined volume of both parotid glands to < 20 Gy or at least 50% of one gland to <30 Gy.

### Treatment planning and radiotherapy

#### Carbon ion therapy

Carbon ion therapy is given at the HIT (Heidelberg ion therapy centre) after inverse treatment planning in active beam application (raster-scanning method) [11]. The PTV is divided into iso-energetic slices roughly corresponding to their radiological depths. The raster-scanning method uses mono-energetic carbon ion beams, which are extracted from the accelerator system (synchrotron) and magnetically deflected so as to scan each of these iso-energetic slices. Using this method, almost any desired dose distribution can be created and dose to surrounding critical structures can be minimized.

Inverse treatment planning is carried out on a dedicated Siemens treatment planning system (TPS®). Due to increased biological effective dose of ion beams TPS® additionally includes methods for inverse treatment planning and biological RT treatment optimization for particle therapy.

Daily image guidance consists of orthogonal x-ray controls in treatment position with robot-mounted x-ray tubes/ receptors. After acquisition of orthogonal x-rays, the automatic 2D-3D pre-match is carried out (Siemens syngo PT treatment) and verified by the radiotherapist/radiation oncologist. Manual adjustment of the match can be carried out on-line and the resulting correction vector, including rotations, is subsequently applied to the patient position. Patient position is controlled at each session and shifts are always corrected using a robot-mounted treatment table allowing position correction in six degrees of freedom.

#### IMRT

IMRT is given at the Dept of Radiation Oncology in 25 fractions (5 fractions per week) either on a dedicated 6 MV tomotherapy unit or on a 6 MV linear accelerator in step and shoot technique after inverse planning either with the optimization tool KonRad MRC® (Siemens OCS) or the dedicated tomotherapy inverse planning software. In both cases, regular image guidance is carried out. If necessary, daily pretreatment online correction of translational vectors is applied. Total doses take account of the doses applied by daily image guidance with MV-cone-beam CT. Standard 3D treatment was not allowed within the protocol.

#### Follow-up

Regular follow up is carried out 6 weeks post treatment, 3 months (4–5 months post completion of therapy) thereafter, and then in 6 monthly intervals including fibreoptic examination and local imaging with MRI.

#### Study design and analysis

COSMIC is a prospective, mono-centric phase II trial evaluating acute mucositis ≥ CTC°3 as the primary endpoint. Planned accrual is 54 patients.

Secondary endpoints are local control (LC), progression-free survival (DFS), and toxicity (acute and late radiation effects). Toxicity is assessed using NCI CTC v 3.0 at treatment completion as well as at each follow-up visit.

Evaluation of disease response is carried out according to the Response Evaluation Criteria in Solid Tumors (RECIST) [12] 6 weeks, 4–5 months, 6–7 months post completion of treatment and then in 6-monthly intervals. Further details can be found in the published trial protocol [10].

## Results

Between July 2010 and April 2011, twenty-nine patients were accrued to the COSMIC trial. No patient was excluded or had to discontinue therapy. All patients completed treatment as scheduled.

Thirteen patients with macroscopically complete resections (group R1) and 16 patients with either incomplete resection (7 pts) or inoperable tumors (9 pts) (group R2) were included. Median age was similar in both groups: 56 years (R2) and 55 years (R1). Most patients had adenoid cystic carcinoma (R2: 16/16 pts, R1: 8/13 pts) and only 5 patients in the R1-group had other histologies. The R2 group included a high number of MSGTs in the paranasal sinus, whereas there was a higher proportion of MSGTs of the large salivary glands in the R1-group. While many patients in the R2-group had very advanced disease (T4: 11 pts), tumor extent in the R1-group tended to be smaller (T4: 6 pts). For one patient in the R2-group a valid TNM staging for tumors of the external auditory canal does not exist, for two patients in the R1-group the pathological TNM stage is unknown. Three patients in the R2 group had known pulmonary metastases at presentation; none of the resected patients in R1 had distant metastases. Details of patients’ baseline characteristics can be found in Table 1.

**Table 1:** Patient baseline characteristics

	**R2: visible residual tumor**	**R1: R1/ Pn + resected tumors**
**Patient number**	16	13
Prior surgery	7	13
Recurrent tumors	3	2
Median age (years)	56	55
Range (years)	27-74	25-72
**Stages**		
T1	0	0
T2	2	1
T3	2	4
T4	11	6
Tx	1	2
N0	13	9
N1	2	0
N2b	1	2
M1	3	0
**Histology**		
Adenoid cystic carcinoma	16	8
Mucoepidermoid carcinoma	0	3
Adenocarcinoma	0	1
Squamous cell carcinoma	0	1
**Site**		
Base of skull	0	1
Paranasal sinus	7	3
Palate	0	1
Nasopharynx	3	0
External auditory canal	1	0
Parotid gland	3	2
Submandibular gland	1	4
Lacrimal gland	1	1
Lacrimal duct	0	1

In all cases, treatment dose could be applied according to protocol (Table 2) and dose prescription recommendations (<5% of CTV1 or 2 receiving less than 90% of the dose prescribed) were met in all patients. While CTV2 volumes are comparable in both groups, median CTV1 volume is much larger in the R2 group. The proportion of patients requiring more complex planning procedures such as intensity-modulated particle therapy (IMPT) remains essentially the same in both groups, while more patients in the R2 group underwent tomotherapy rather than step-and-shoot IMRT and more patients (R2: 6 pts; R1: 2 pts) underwent bilateral nodal irradiation in the R2 group (Table 2).

**Table 2:** Treatment characteristics

	**R2: visible residual tumor**	**R1: R1/ Pn + resected tumors**	**total**
**Patient number**	16	13	29
**Median dose (GyE/ Gy)**	74.5	74.2	74.2
C12	23.81	23.87	23.83
IMRT	50.74	50.42	50.6
**C12 optimization technique**			
IMPT (pts)	11	9	20
SBO (pts)	5	4	9
**IMRT technique**			
step& shoot IMRT	10	12	22
Tomotherapy	6	1	7
**Treatment volumes (ml)**			
CTV1	167.1	94.13	138.38
CTV1 range	161.95 - 618.5	48.66 - 187.82	32.56 - 390.6
CTV2	466.73	413.2	431.5
CTV2 range	190.13 - 922.71	32.56 - 390.6	161.9 - 922.7
Bilateral level II/III (pts)	6	2	8

### Treatment tolerance and toxicity

Treatment was well tolerated although treatment including set-up, position verification, and irradiation for carbon ions takes between 30 and 45 min.

Treatment-related acute effects as assessed at completion of radiotherapy were generally mild with mucositis CTC °III occurring in 25% (R2) and 15.4% (R1). Despite comparatively extensive treatment fields, only mild xerostomia was observed; at completion of treatment, rates of xerostomia CTC°II were 15.4% in the R1 group and none in the R2 group. Almost all (100% in R2 and 84.6% in R1) reported severe to complete loss of taste by the end of treatment leading to weight loss in 81.3% (R2) and 69.2% (R1) of patients. There was no higher-grade dysphagia, only few patients (R1: 15.4%) reported dysphagia CTC°II. None of the patients required a feeding tube. However, due to dysgeusia and subsequent loss of appetite, we found a median weight loss of 5 kg (R2) and 5.5 kg (R1) in our patient cohort. Two patients in the R2 group and one patient in the R1 group showed reduced jaw opening prior to and at completion of therapy: one patient improved under therapy due to tumor regression. Roughly 37.5% of patients in R2 developed middle ear effusions during therapy and only 15.4% in R1.

Symptoms rapidly resolved after treatment, only one patient in the R2-group showed mucositis CTC°I at first follow-up (6–8 weeks post completion of treatment), otherwise there was no case of residual mucositis. Xerostomia was reported at an overall rate (CTC°I/II) in 75% (R2) and 84.6% (R1), only 12.5% of patients in the R2-group reported mild swallowing difficulties on their first follow-up appointment. All patients reported improvement of dysgeusia with dysgeusia slowly resolving with time and leading to further weight loss post completion of treatment in 18.8% (R2) and 30.8% (R1) (Table 3). However, body weight had stabilized in all of these patients on their first follow-up.

**Table 3:** Adverse events

		**R2: visible residual tumor**		**R1: R1/ Pn + resected tumors**	
		**end of RT**	**1st f/u**	**end of RT**	**1st f/u**
**Mucositis**	I	3 (18.8%)	1 (6.3%)	4 (30.8%)	0
	II	7 (43.8%)	0	6 (46.2%)	0
	III	4 (25%)	0	2 (15.4%)	0
**Dermatitis**	I	13 (81.3%)	0	12 (92.3%)	1 (7.7%)
	II	2 (12.5%)	0	0	0
	III	1 (6.3%)	0	1 (7.7%)	0
**Xerostomia**	I	7 (43.8%)	10 (62.5%)	6 (46.2%)	11 (84.6%)
	II	0	2 (12.5%)	2 (15.4%)	0
**Dysphagia**	I	7 (43.8%)	2 (12.5%)	4 (30.8%)	0
	II	0	0	2 (15.4%)	0
	III	0	0	0	0
**Weight loss**	yes	13 (81.3%)	3 (18.8%)	9 (69.2%)	4 (30.8%)
**kg**	median	5		5,5	
	min	3		5	
	max	10		12	
**Feeding tube**		0	0	0	0
**Loss of taste**		16 (100%)	0	11 (84.6%)	0
**Middle ear effusion**		6 (37.5%)	5 (31.3%)	2 (15.4%)	2 (15.4%)
**Otitis**		0	0	0	0
**Paralysis of facial nerve**		1 (6.3%)	1 (6.3%)	1 (7.7%)	1 (7.7%)
**Ptosis**		1 (6.3%)	1 (6.3%)	0	0
**Reduced jaw opening**		2 (12.5%)	2 (12.5%)	1 (7.7%)	1 (7.7%)
**Xerophthalmia**		0	1 (6.3%)	1 (7.7%)	0
**Conjunctivitis**		1 (6.3%)	0	0	0
**Lymph edema**		1 (6.3%)	1 (6.3%)	0	2 (15.4%)
**Hearing impairment**		2 (12.5%)	0	0	0

### Treatment response

**Figure 1:**
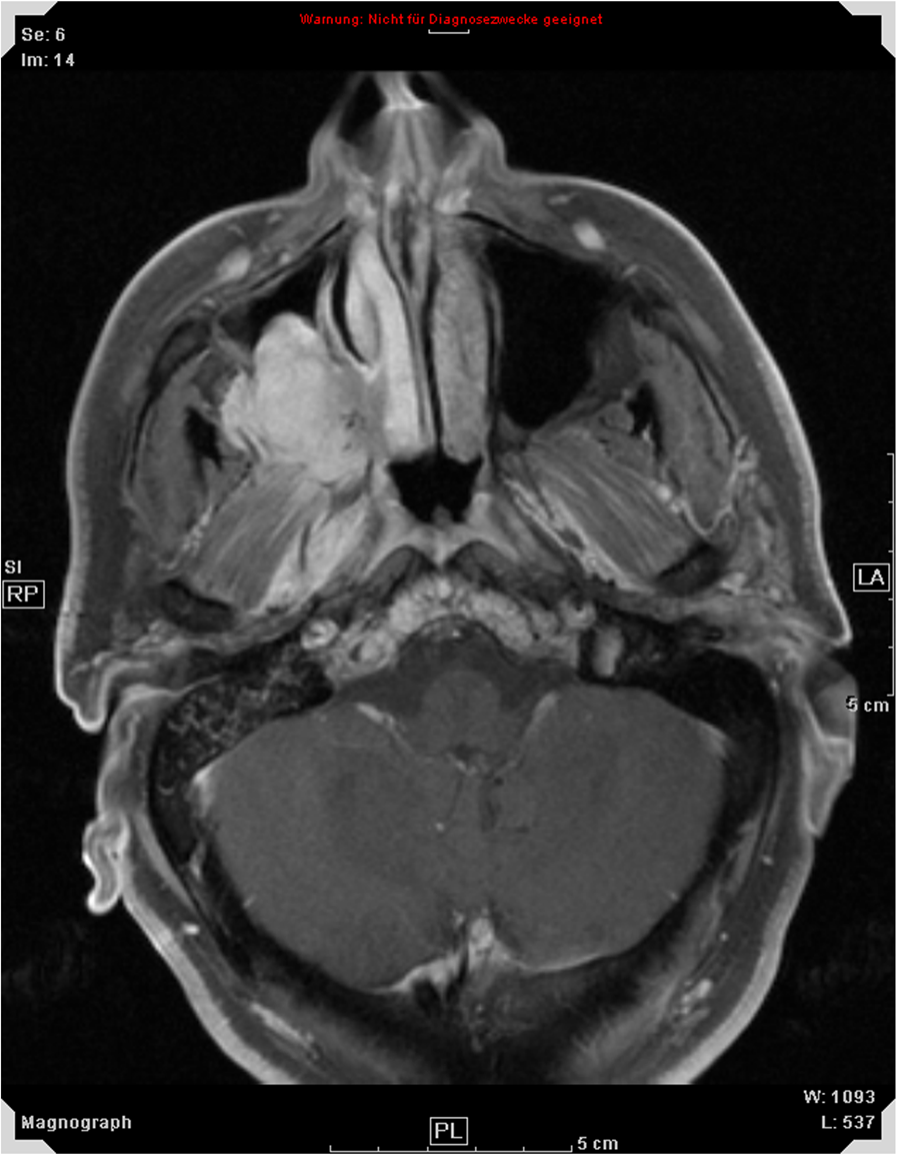
51 year old patient with adenoid cystic carcinoma extending from the right maxillary sinus into the right orbit and cavernous sinus, contrast-enhanced, T1-weighted MRI for treatment planning.

**Figure 2:**
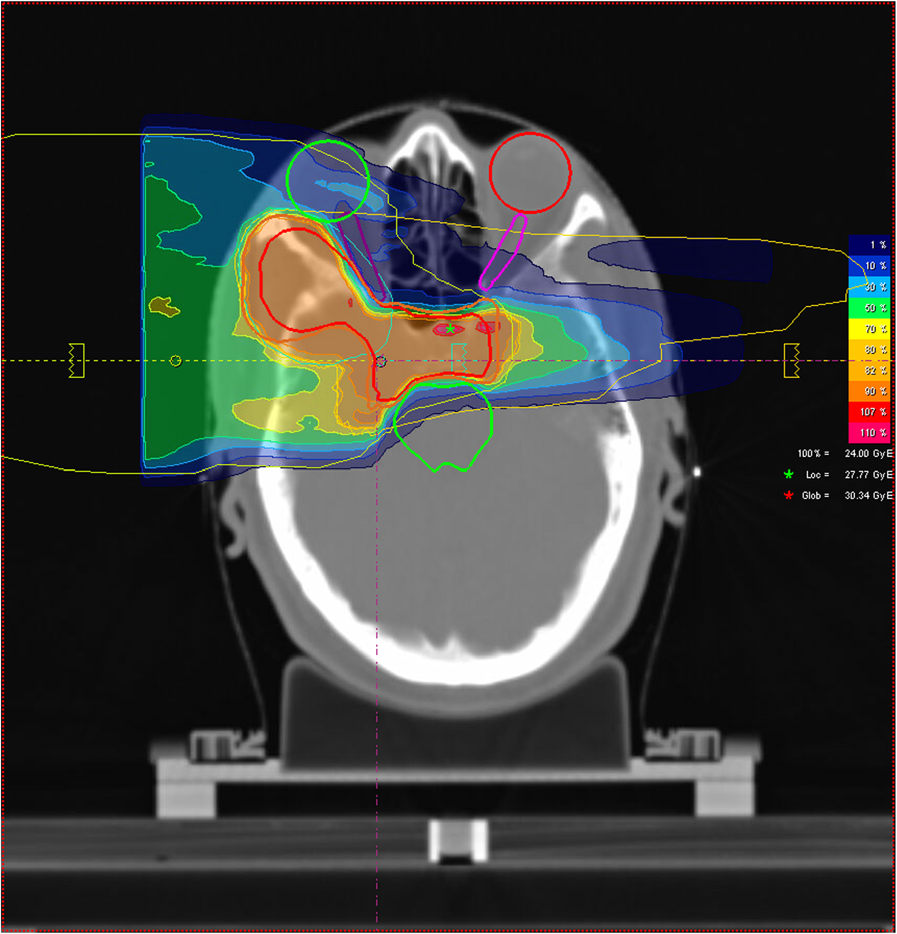
Axial carbon ion dose distribution (orbit/ cavernous sinus), 3-field IMPT, 100% corresponding to 24 GyE carbon ions.

**Figure 3:**
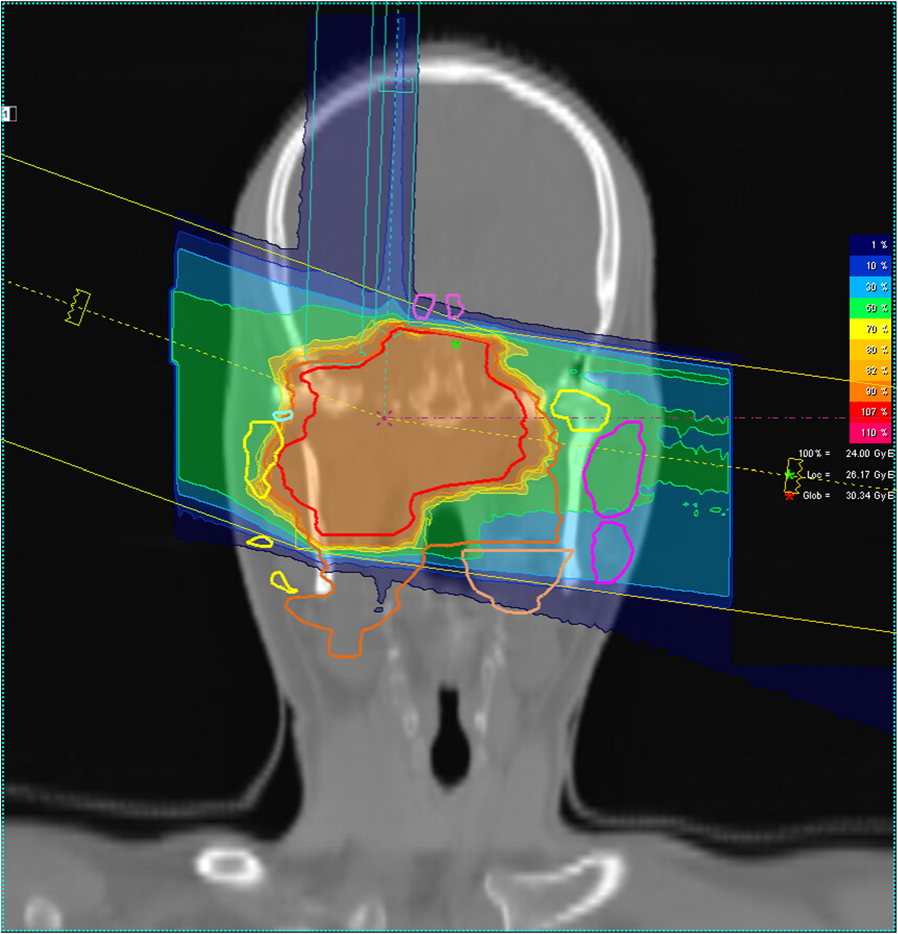
Coronal carbon ion dose distribution, 3-field IMPT, 100% corresponding to 24 GyE carbon ions.

**Figure 4:**
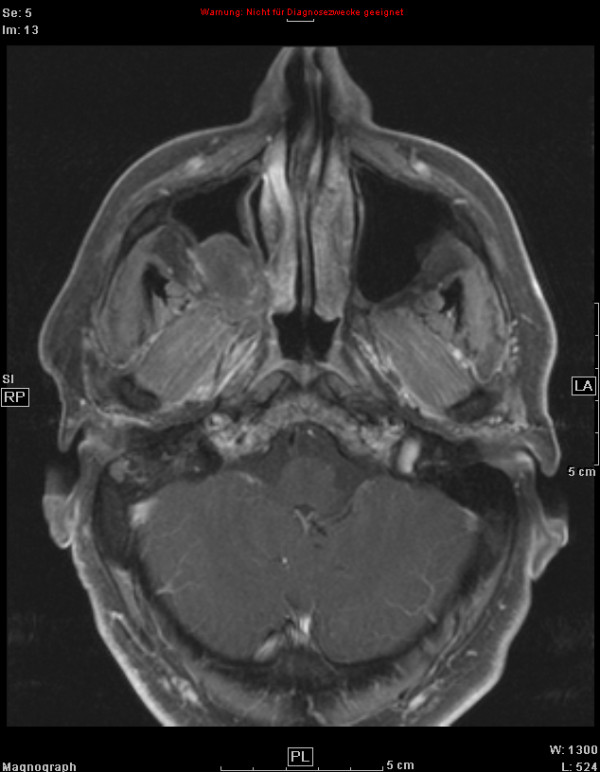
51 year old patient with adenoid cystic carcinoma, contrast-enhanced, T1-weighted MRI at first follow-up showing partial remission but highly reduced contrast-enhancement.

Overall local response rate (complete and partial remissions) 6–8 weeks after completion of treatment in the R2 group is 68.8% (CR: 2/16 pts, PR: 9/16 pts, SD: 5/16 pts) according to RECIST. However, all of the patients showed signs of tumor response such as reduced contrast-enhancement on MRI, carbon ion dose distribution, initial and follow-up MRI scans of a patient with good PR is shown in Figures 1, 2, 3, 4. With our median follow up of 3 months [range 3–12 months], one of the partial remissions has already developed into a complete remission 6 months post treatment.

One patient with adenoid cystic carcinoma and pulmonary metastases in the R2-group unfortunately showed a very good locoregional PR but distant disease progression and is currently undergoing palliative chemotherapy. There was no other case of disease progression in either the R2 or the R1 group.

## Discussion

Introduction of high precision radiotherapy and particle therapy has changed patients prognosis in this rare condition. Neutron radiotherapy was able to achieve high local control rates in the past [2,13,14], the RTOG-MRC randomized trial reported a 67% local control in the neutron group as opposed to 17% in the photon group in unresectable MSGTs [13]. However, this treatment was often accompanied by significant side effects such as temporal lobe necrosis, ulceration, spinal cord myelopathy, and loss of vision due to optic neuropathy, retinopathy, acute angle glaucoma and bleeding [2,14]. Severe long-term effects were reported in more than 14% of patients [2].

Recent results in charged particle therapy produced much more favorable toxicity profiles even though hypofractionated, dose-escalated regimen were used [4-6,15]. No CTC°III late toxicities and very few °III acute reactions occurred in the Japanese treatment regimen published by Mizoe et al applying 70.2 GyE (3 × 3.9 GyE/wk) or 64 GyE (4 × 4 GyE/ wk) [4]. Including various histological subtypes of MSGTs, local control at 5 years was 100%. Pommier et al. [15] treated 23 patients with adenoid cystic carcinoma with protons at 75.9 GyE (median) in various fractionation schemes. Overall local control at 5 years was 93%, however, there was one °V late toxicity (temporal lobe necrosis).

IMRT (54 Gy) plus carbon ion boost (18 GyE à 3 GyE) at a biologically equivalent dose of roughly 77 Gy for adenoid cystic carcinoma however, also achieved impressive results without any higher-grade (CTC°III or higher) late toxicity resulting in local control rates of 78% in 4 years [5] with a recent update confirming these results [6]. Predominant site of relapse in this cohort is still in-field or within the dose gradient to adjacent organs at risk despite relative already high radiation doses. Based on this experience, COSMIC includes a further dose escalation mainly of the carbon ion part to 24 GyE (à 3 GyE per fraction) and IMRT to 50 Gy corresponding to a biologically equivalent dose of approximately 80 Gy with the aim to further improve treatment results [10]. In addition, COSMIC is the first trial to include patients without visible residual tumor. Hence potential side effects may be increased. COSMIC is also recruiting uncommonly fast in this very rare disease, therefore an interim analysis to exclude unexpected higher-grade toxicity is necessary.

### Toxicity

To present, no unexpected acute toxicity has occurred in the COSMIC patients with transient alteration of taste, mild xerostomia, and dysphagia being the most frequently reported. Also, we found no higher grade (CTC°III or higher) acute toxicity which is in good agreement with other charged particle therapy series [4,5,15].

Treatment volumes tended to be large (CTV1: 138.38 ml, CTV2: 431.5 ml), even though, there was no case of CTC°III dysphagia and only 6/29 patients with mucositis CTC°III (20.7%; R2: 25%, R1: 15.38%). Patients in the R1 group did not show a higher percentage of grade III mucositis as compared to patients with visible residual disease. Although mucositis CTC°III was reported in only 6% of patients in our previous series [5,16], however, this series predominantly included tumors located at the base of skull, no patients with tumors of the major salivary glands were included. Mucositis rates in the Japanese series were also comparatively low, but target volumes also tended to be much smaller [4]. R1-resected tumors not including the skull base were not included in both cohorts [4,5]. In view of treated anatomical sites, occurrence of CTC °III mucositis at roughly 20% is still low especially considering the fact that our cohort included 10 patients with tumors of the major salivary glands and 10 patients with extensive tumors of the paranasal sinuses.

Perineural infiltration and skip lesions are significant, independent predictors of local control in most series for MSGTs [7,8,17,18], therefore our policy is to include potentially involved neural tracts up until their entrance into the skull base as proposed by Garden et al [8]. For the facial nerve, this brings higher dose close to the middle ear therefore, the occurrence of middle ear effusions in 8/29 patients (27.6%) is not surprising. In all cases, these symptoms were at least improving at first follow-up and are therefore acceptable.

### Efficacy

Follow-up is yet too short to analyze efficacy in the R1 group. However, response rates and extent of response in the R2 group are promising: overall response (CR and PR) was 68.8% with 2/16 pts showing complete remissions according to RECIST at the first follow-up. All of the patients in the R2 group showed significantly reduced contrast-enhancement on their follow-up MRI. Very little data has been published on response rates and timeline of response in MSGTs. Our own experience suggests high response rates of patients with adenoid cystic carcinoma with the tumor slowly regressing and disappearing within time spans between 6–12 months. Data from the neutron era report tumor clearance rates of up to 84.6% (11/13 pts) but does not mention time intervals [13]. The authors do see a correlation between tumor response and long-term local control, therefore our response rates at a median follow-up of 3 months seems promising.

Some limitations of the trial have shown up in this interim analysis:

Approximately 13 of 29 patients underwent macroscopically complete resections and exhibit risk factors such as involved margins or perineural spread (R1), and 16/29 patients still had visible residual tumor (R2), the two cohorts are comparable in a first approximation. However, COSMIC should include various types of MSGTs in order to establish the carbon ion combination regimen for MSGT histologies other than adenoid cystic carcinoma. Most patients recruited so far were diagnosed with adenoid cystic carcinoma, further investigations need to exclude adenoid cystic carcinoma from the trial to address this issue.

Dose-escalated combination treatment may hopefully lead to even higher local control rates, however, none of the local treatment regimens has yet had an impact on overall survival or distant metastasis-free survival in malignant salivary gland tumors [5,7,8,19]. The use of concomitant chemotherapy or immunotherapy in squamous cell carcinoma of the head and neck (SCCHN) [20,21] has led to a significant improvement not only in local control but also in overall survival. Radiochemotherapy in treatment of malignant salivary gland tumors however, has not evolved beyond the phase II-stage or retrospective analysis of very heterogeneous treatment regimen [22-24] into a treatment standard so far.

Hence another questions remains: patients with adenoid cystic carcinoma may also benefit from combined treatment with i.e. new substances such as EGFR-inhibitors in terms of local and distant control [25]. However, to our knowledge, there is no prospective clinical trial evaluating combination treatment of any substance with carbon ion therapy, hence we will shortly commence a phase-II trial with the EGFR-receptor antibody cetuximab addressing this issue [26].

## Conclusion

There is no unexpected toxicity in the COSMIC trial, mucositis rates and other side effects do not seem to differ in the patients without visible residual tumors (R1 group). Initial treatment response of the dose-escalated regimen is promising. In view of a median follow-up of 3 months in our patients, longer follow-up is needed to assess secondary trial endpoints such as local control and progression-free survival.

## Competing interests

The authors declare that they have noc competing interests.

## Authors’ contributions

ADJ, JD, and MWM were responsible for concept and design of the trial; ADJ, AVN, KKH, and MWM were responsible for patient accrual and treatment. ADJ, AVN, and KL coordinated and organized the trial. All authors read and approved the final manuscript.

## Pre-publication history

The pre-publication history for this paper can be accessed here:

http://www.biomedcentral.com/1471-2407/12/163/prepub
